# Tobacco cues in India: An ecological momentary assessment

**DOI:** 10.1186/s12971-016-0081-z

**Published:** 2016-05-04

**Authors:** Dina L. G. Borzekowski, Julia Cen Chen

**Affiliations:** Department of Behavioral and Community Health, School of Public Health, University of Maryland, #2364 SPH Building, Valley Drive, College Park, MD 20742 USA

**Keywords:** Ecological momentary assessment, Tobacco use, Social and environmental cues, Regulations, India

## Abstract

**Background:**

Tobacco use in India is a major health concern; however, little is known about the influence of tobacco-related social and environmental cues on tobacco use. This study uses ecological momentary assessment (EMA) to examine real-time tobacco use and exposure to social and environmental cues.

**Methods:**

In Hyderabad and Kolkata, participants were recruited, and an EMA application was installed on their mobile phones. Momentary prompts (MP) were randomly used to collect real-time information and end-of-day (EOD) prompts gathered retrospective information on daily basis. Besides personal tobacco use, the surveys asked about exposure to social (e.g., presence of others using tobacco) and environmental cues (e.g., visual and olfactory stimuli). Using the data aggregation approach, bivariate and multivariate analyses were performed to examine the association of tobacco use and cue exposure. Moderating roles of participants’ socio-demographic characteristics were also tested to gain an in-depth understanding of the relationship.

**Results:**

Among the 205 participants, around a third (MP, 33.7 %; EOD, 37.6 %) used tobacco at least once during the study period. Tobacco-related social and environmental cues related were commonly reported. In the bivariate models, tobacco use was associated with gender, age, and all the examined social and environmental cues except for seeing restrictions on tobacco use. In the multivariate models, tobacco use was associated with age, gender, seeing others using tobacco, and seeing restrictions on tobacco use. Seeing others in one’s immediate group using tobacco was the strongest predictor of tobacco use in both MP and EOD assessments. Gender and age did not moderate the relationship between cue exposure and tobacco use, although males reported higher tobacco use and cue exposure in general.

**Conclusions:**

This research provides data on the ubiquity of social and environmental tobacco cues in India. The EMA approach was feasible and informative. Future cessation interventions and advocacy efforts should address the high prevalence of tobacco use and exposure to pro-tobacco use cues especially among Indian males. Health education campaigns for promoting tobacco use restrictions in private places as well as changing the norms of tobacco use in social settings are recommended.

## Background

In India, tobacco use is a major risk factor for numerous chronic and severe health problems, including cardiovascular disease, obstructive pulmonary disease, and cancer [1–3]. According to the World Health Organization (WHO) Global Adult Tobacco Survey (GATS) data from 2009, tobacco use was nearly 50 % for males and 20.3 % for females in India [4]. Despite the overwhelming direct and indirect toll of tobacco use, relatively little research has been conducted in India studying exposure to social and environmental cues associated with tobacco uptake and real-time behaviors. Without current valid and reliable information, it is hard to understand initiation and continued use of tobacco. Knowing the frequency and location of cues can aid prevention and cessation efforts in India. Additionally, advocacy and regulatory efforts would benefit from better data.

Numerous studies in western countries document links between social and environmental cues and tobacco use and behaviors. In the U.S., smoking is more likely to occur during social occasions and around bars and restaurants [5] or during breaks between formal activities; [6] by contrast, tobacco use is significantly less likely to happen when people are engaged in structured, work-related activities [5]. Others’ smoking can stimulate craving for tobacco use [7, 8] or actual tobacco use, [5] mainly by providing visual and olfactory sensory cues [9, 10]. Additionally, perceived social norms around use tobacco products can encourage smoking [11, 12]. The research is mixed as to whether it makes a difference if one thinks “others” who are smoking are known acquaintances or strangers [13, 14].

Environmental cues also influence tobacco use. Laboratory studies show that visual and olfactory stimuli can increase cardiovascular activation, including blood pressure and heart rate, and directly prompt tobacco use [9, 15–17]. Additionally, pro- and anti-tobacco messages delivered through media (i.e., newspapers, radio, TV, and the Internet, etc.) affect tobacco behaviors [18–20]. Considerable evidence indicates that cigarette advertising is related to increased tobacco consumption, especially among teenagers and young adults [20, 21]. One U.S. study of real-time exposure to pro-tobacco marketing and media found that exposure was significantly associated with greater current and future smoking intentions [19]. Reported smoking restrictions is another important environmental exposure influencing craving for smoking [10] and smoking [5]. Researchers have found the odds of smoking were reduced by 64 % when smoking was forbidden; conversely, the odds were increased 119 % when smoking was allowed [5]. A cross-sectional study found that smoking restrictions were related to lower smoking prevalence at home, public places and schools [22].

A major concern with tobacco research is that retrospective data is not just subject to random error but also is fraught with systematic bias, which can distort recall even after relatively short intervals [23]. For example, reported experiences are more likely if the experience is emotionally salient or unique; routine experiences are less likely to be reported [24]. An alternative approach to assessing situational or contextual associations with public health behaviors is Ecological Momentary Assessment (EMA) [24]. EMA collects real-time data about participants’ natural environments. Two approaches of EMA include fixed and random prompts. The random approach involves signaling participants at different times throughout the day. With the fixed approach, assessments are expected and regular (e.g., once a day at noon) [24]. Although the fixed approach still requires a brief period of recall, it may serve as a proxy for the random approach [25].

Published EMA studies have explored associations of immediate and predictive antecedents, as well as contextual factors, with smoking behaviors [19, 26–29]. These studies, however, have only been done in the U.S. and other developed countries. Our study is the first to be done in a lower- or middle-income country (LMIC). In the Indian cities of Hyderabad and Kolkata, we collected data using the two aforementioned EMA approaches. Amongst a sharp worldwide increase of mobile phone penetration over the past decade, mobile phones are a practical way to collect real-time public health data. India had more mobile phone subscriptions in 2011 than Africa, the Middle East, and Europe—72 out of every 100 inhabitants in India have a mobile/cellular subscription [30]. Adults aged 18 to 24 years also make up a higher-than-average proportion of mobile phone users at 13 % [30].

Our objective for this work was to identify exposure to social and environmental tobacco-related cues and examine associations with demographic and tobacco use experience. We hypothesized that participants’ exposures to social and environmental cues differ by gender, age, employment, education, as well as their tobacco use status. The purpose of such work was to inform future research and advocacy efforts in India and other LMICs.

## Methods

### Participant recruitment and training

Participants were recruited through schools and colleges, work offices, and popular neighborhood places in Hyderabad and Kolkata, India. Inclusion criteria for the study included possession of an Android-series mobile phone, or one with similar functions and application capabilities. During recruitment, an effort was made to recruit a sample where half the participants, both males and females, are tobacco users, in order to have an adequate number of participants from both groups. All participants were adults (over 18 years of age) and provided written consent to join the study. The study was approved by Institutional Review Boards at the Johns Hopkins Bloomberg School of Public Health and the University of Maryland, as well as from the Biomedical Ethics Committee in India. After recruitment, a training session was held to familiarize the participants with the study procedures. Researchers installed the EMA application (app) on the participants’ mobile phones and time was allotted for practicing with the app. Participants received a month of unlimited data for their phones as well as a flash drive at the completion of the study. The app recorded incomplete or timed-out surveys as “expired” in the dataset and did not record partial responses. If participants turned off their phones or if phones were in a poor reception area, the app would mark data as missing or display blank cells in the dataset.

### Study procedures

From January to May 2014, in staggered time periods, participants were randomly signaled 5–8 times per day for ten consecutive days during waking hours (defined as 8 am to 10 pm) on their mobile phone, which instructed them to take the momentary prompt (MP) survey. The end of day (EOD) survey was programmed to send at 10 pm each day during the study period [5, 24, 31]. The same set of questions in the two surveys was used each time, though the order of MP questions changed to counterbalance the effects of item sequencing [24] and prevent response fatigue. Participants had 30 min to respond to a prompt once they started; the dataset would mark it as expired if they did not finish in this time. If participants were unable to use their phone when signaled, for example during a meeting or while driving, they could put the app on hold or “snooze” for up to 20 min. After 20 min, the participant could no longer take that particular survey and it would be sent back as expired. All surveys were programmed to send to the users automatically, and participants could not manually or self-initiate a prompt.

### Measures

The MP and EOD surveys included multiple-choice questions adapted from similar EMA questionnaires typically done in the U.S., [32] combined with adapted portions of the WHO Global Adult Tobacco Survey (GATS) [2]. Baseline characteristics included: 1) Sex (Male, Female), 2) Education Level (< College degree, ≥ College degree), 3) Employment Status (Unemployed, Employed), and 4) Household Car Ownership (No, Yes). Age (in years) was used a continuous variable. Since tobacco is consumed in various forms in India, this study included questions about smoked and smokeless tobacco [33, 34]. To answer real-time tobacco use, participants responded to the MP question “Are you currently using a tobacco product?” The EOD survey asked “Did you use any tobacco products today?” Participants could answer “no”, “yes, smoke tobacco”, or “yes, smokeless tobacco” in both assessments. The MP and EOD surveys asked about potential exposures associated with tobacco use. Social exposure included presence of other tobacco users (no, others in one’s group, and other’s in one’s sight). Environmental exposures were the sight of others using tobacco, the visual and olfactory cues of tobacco use, the sight of pro-and anti-tobacco messages, and known restrictions of tobacco use. The MP assessment also captured participants’ locations and companionship at the time they were signaled. Locations included private places (i.e. homes and cars), work/school, public setting (e.g., restaurants, bars, stores, places of worship, etc.), and outside. Companionship responses included alone, with family, and with friends/coworkers/strangers. The EOD assessment did not capture the measures location and companionship, and for the social cue presence of other tobacco users, it only allowed the answer choices of “yes” or “no.”

### Data analysis

On average, the resulting completion rates for MP and EOD were 0.47 (SD = 0.21) and 0.73 (SD = 0.27), respectively. Participants spent 3.84 (SD = 2.21) and 3.62 (SD = 2.82) minutes to complete the MP and EOD surveys, respectively. Analyses were performed on these completed MP and EOD reports (5,603 for MP survey and 988 for EOD survey) and the expired prompts (3,477 for MP survey and 124 for EOD survey) were dropped from the final analysis as all of the responses were missing. A separate paper describes with great detail the feasibility of EMA with this sample, exploring what predicted compliance [35]. It was found that employment status was the only predictor of MP or EOD compliance. Based on this work, researchers concluded that EOD assessments might be preferable, as they involved lower researcher and participant burden and were consistently correlated with MP responses [35].

For this study, the unit of analysis was individual participant. Participants were considered tobacco users if in either the MP or EOD assessments they reported using tobacco *anytime* during the 10-day study period. Similar methods were used to measure social and environmental exposures —participants were considered having these exposures if they reported these exposures anytime during the study, in either MP or EOD assessments. Student *t*- and chi-square tests were used to analyze 1) the differences of social and environmental exposures between sex, age, education, and income; and 2) the association of EMA tobacco use status with reported social and environmental cues. Significance level was set at 0.05. Data analysis was performed using SPSS 22.0 [36].

## Results

Table 1 offers information about the sample of 205 participants broke down by tobacco use status according to MP and EOD assessments. Males (40.7 %) were more likely to use tobacco than females (20.0 %) according to the MP assessment (*X*^2^ (df = 1) = 8.88, *p* < 0.01), and older participants (M = 26.3, SD = 6.8) were more likely to use tobacco than younger participants (M = 24.2, SD = 5.6) according to the EOD assessment (t (203) = -2.47, *p* < 0.05). About 33.7 % and 37.6 % (*N* = 69 and 77) of the participants used smoke or smokeless tobacco over the period of assessment according to the MP and EOD surveys, respectively. Table 2 presents information about various tobacco-related cues reported through the MP and EOD assessments.

**Table 1:** Baseline characteristics and exposure to social and environmental cues by tobacco use, MP and EOD assessments (*N* = 205)

	MP tobacco use				EOD tobacco use			
	Total	No	Yes		Total	No	Yes	
		*N* (%)	*N* (%)	*P*		*N* (%)	*N* (%)	*P*
		136 (66.3)	69 (33.66)			128 (62.4)	77 (37.56)	
Age^a^	24.99 (6.15)	24.81 (6.07)	25.35 (6.33)	0.55	Age^a^	24.99 (6.15)	26.34 (6.80)	< 0.05
Sex								
Male	135 (65.85)	80 (58.82)	55 (79.71)	< 0.01	135 (65.85)	79 (61.72)	56 (72.73)	0.11
Female	70 (34.15)	56 (41.18)	14 (20.29)		70 (34.15)	49 (38.28)	21 (27.28)	
Education level								
< College degree	133 (65.84)	89 (66.92)	44 (63.77)	0.65	133 (65.84)	88 (69.84)	45 (59.21)	0.12
≥ College degree	69 (34.16)	44 (33.08)	25 (36.23)		69 (34.16)	38 (30.16)	31 (40.79)	
Employment status								
Employed	58 (28.29)	34 (25.00)	24 (34.78)	0.27	58 (28.29)	32 (25.00)	26 (33.77)	0.34
Unemployed	28 (13.66)	21 (15.44)	7 (10.14)		28 (13.66)	17 (13.28)	11 (14.29)	
Student	119 (58.05)	81 (59.56)	38 (55.07)		119 (58.05)	79 (61.72)	40 (51.95)	
Car ownership^b^								
No	78 (39.00)	51 (38.06)	27 (40.91)	0.70	78 (39.00)	50 (39.68)	28 (37.84)	0.80
Yes	122 (61.00)	83 (61.94)	39 (59.09)		122 (61.00)	76 (60.32)	46 (62.16)	
Saw others using tobacco (in one’s group)								
No	154 (75.12)	127 (93.38)	27 (39.13)	< 0.001	106 (51.71)	93 (72.66)	13 (16.88)	< 0.001
Yes	51 (24.88)	9 (6.62)	42 (60.87)		99 (48.29)	35 (27.34)	64 (83.12)	
Saw others using tobacco (in one’s view)								
No	117 (57.07)	93 (68.38)	24 (34.78)	< 0.001	88 (42.93)	66 (51.56)	22 (28.57)	< 0.01
Yes	88 (42.93)	43 (31.62)	45 (65.22)		117 (57.07)	62 (48.44)	55 (71.43)	
Saw visual cues of tobacco use								
No	86 (41.95)	68 (50.00)	18 (26.09)	< 0.01	65 (31.71)	57 (44.53)	8 (10.39)	< 0.001
Yes	119 (58.05)	68 (50.00)	51 (73.91)		140 (68.29)	71 (55.47)	69 (89.61)	
Smelled olfactory cues of tobacco use								
No	42 (20.49)	42 (30.88)	0 (0.00)	< 0.001^*^	61 (29.76)	54 (42.19)	7 (9.09)	< 0.001
Yes	163 (79.51)	94 (69.12)	69 (100.00)		144 (70.24)	74 (57.81)	70 (90.91)	
Saw pro-tobacco messages								
No	89 (43.41)	73 (53.68)	16 (23.19)	< 0.001	150 (73.17)	110 (85.94)	40 (51.95)	< 0.001
Yes	116 (56.59)	63 (46.32)	53 (76.81)		55 (26.83)	18 (14.06)	37 (48.05)	
Saw anti-tobacco messages								
No	48 (23.41)	38 (27.94)	10 (14.49)	< 0.05	85 (41.46)	70 (54.69)	15 (19.48)	< 0.001
Yes	157 (76.59)	98 (72.06)	59 (85.51)		120 (58.54)	58 (45.31)	62 (80.52)	
Saw restrictions of tobacco use								
No	24 (11.71)	13 (9.56)	11 (15.94)	0.179	Not Available			
Yes	181 (88.29)	123 (90.44)	58 (84.06)					

^*^The significance level was obtained from the Fisher’s exact test due to small data cell

^a^Age (years) reported in mean and standard deviation (range = 18 to 38, medium years = 22)

^b^ *N* = 200; five missing

**Table 2:** Multivariate analysis of tobacco use by baseline characteristics and cue exposures, MP and EOD assessments (*N* = 205)

	MP tobacco use^a^			EOD tobacco use^b^		
	AOR	CI	*P* value	AOR	CI	*P* value
Age	0.94	0.85–1.03	0.16	1.11	1.01–1.21	< 0.05
Sex						
Female	Reference			Reference		
Male	3.10	1.07–8.94	< 0.05	1.91	0.69–5.28	0.22
Education level						
≥ College degree	Reference			Reference		
< College degree	0.69	0.27–1.80	0.45	0.47	0.19–1.13	0.09
Employment status						
Employed	Reference			Reference		
Unemployed	0.36	0.09–1.47	0.16	1.25	0.34–4.63	0.73
Student	0.72	0.24–2.29	0.57	1.52	0.52–4.47	0.45
Car ownership						
No	Reference			Reference		
Yes	0.90	0.38–2.17	0.82	1.48	0.64–3.43	0.36
Saw others using tobacco (in one’s group)						
No	Reference			Reference		
Yes	21.1	7.61–58.74	< 0.001	16.3	5.94–44.82	< 0.001
Saw others using tobacco (in one’s view)						
No	Reference			Reference		
Yes	1.27	0.52–3.11	0.60	4.76	1.79–12.65	< 0.01
Saw visual cues of tobacco use						
No	Reference			Reference		
Yes	3.17	1.25–8.04	< 0.05	1.09	0.33–3.56	0.89
Saw pro-tobacco messages						
No	Reference			Reference		
Yes	2.75	0.97–1.79	0.06	2.24	0.91–5.49	0.08
Saw anti-tobacco messages						
No	Reference			Reference		
Yes	0.50	0.14–1.72	0.27	0.99	0.37–2.60	0.98
Saw restrictions of tobacco use				Not Available		
No	Reference					
Yes	0.27	0.08-0.83	< 0.05			

^**a**^ *R* ^2^ = 0.37

^b^ *R* ^2^ = 0.36

### Results from Momentary Prompts (MP)

A third of the participants (33.7 %, *n* = 69) reported tobacco use when they were promoted through the MP assessment, at least once during the study period. All of the participants who reported tobacco use smoked some form of tobacco and 38 (55.1 %) of them also reported using smokeless tobacco. Among the 69 people who used tobacco, 26 (37.7 %) only reported use at a single MP, 9 (13.1 %) at two prompts, and 34 (49.3 %) at three or more prompts. Similar frequencies appeared for the use of smokeless tobacco. Among those 38 dual users, 11 (28.9 %) of them reported tobacco use at a single MP, 5 (13.2 %) at two prompts and 22 (57.9 %) at 3 or more prompts. Only sex was associated with self-reported use of tobacco; 40.7 % of males compared to 20.0 % of females used tobacco at least once (*Χ*^2^ (df = 1) = 8.9, *p* < 0.01). Tobacco use occurred most frequently when participants were at home or in a car (53.6 %, *n* = 37), less frequently when they were outside or in other places (47.8 %, *n* = 33) or public settings (44.9 %, *n* = 31), and least frequently when at work or at school (40.6 %, *n* = 28). In terms of companionship, tobacco use occurred most frequently when participants were with friends, coworkers and others (71.0 %, *n* = 49) and less frequently when alone (46.4 %, *n* = 32) and least frequently when with families (40.6 %, *n* = 28).

Through MP assessments, about half (48.8 %, *n* = 100) of the sample indicated that they had been in the presence of another tobacco user during the study period. Among those who reported others using tobacco, 34 (34.0 %) of them reported such presence once, and 66 (66.0 %) reported more than once. Presence of other tobacco users was most frequently seen outside or in other places (60.0 %, *n* = 60) and private places (59.0 %, *n* = 59), less frequently in public settings (31.0 %, *n* = 31) and school and workplaces (29.0 %, *n* = 29). Of these 100 participants who reported seeing other tobacco users, half (51.0 %, *n* = 51) of them reported seeing these tobacco users in their immediate group, and most (88.0 %, *n* = 88) reported seeing these tobacco users in their view. Males (31.1 %, *n* = 42) were more likely to be with other tobacco users in their immediate group, compared to females (12.9 %, *n* = 9, *Χ*^2^ (df = 1) = 8.2, *p* < 0.01). A much higher percentage of tobacco users (81.2 %, *n* = 56) reported this social cue compared to non-tobacco users (32.4 %, *n* = 44) (*Χ*^2^ (df = 1) = 43.6, *p* < 0.001).

More than half encountered visual (58.1 %, *n* = 119) and the majority of the participants experienced olfactory (79.5 %, *n* = 163) cues of tobacco use, during the 10 day study period. Among these who encountered visual cues, around 28.6 % of them (*n* = 34) had this exposure just once, while 71.4 % (*n* = 85) of them had this exposure two or more times. Among those reporting olfactory cues of tobacco use, around 23.3 % of them (*n* = 38) had the exposure once, while many more (76.7 %, *n* = 125) had experienced this exposure two or more times. Visual cues were most frequently reported in private settings (62.2 %, *n* = 74), slightly less frequently outside or in other places (52.9 %, *n* = 63), and least frequently in public settings (30.3 %, *n* = 36). Similarly, olfactory cues were most frequently reported in private settings (73.6 %, *n* = 120), less frequently outside or in other places (48.5 %, *n* = 79), and least frequently in public settings (31.3 %, *n* = 51). No demographic variables were associated with reported visual cues, but a significantly higher percentage of tobacco users (73.9 %, *n* = 51) encountered this type of cue than non-tobacco users (50.0 %, *n* = 68, *Χ*^2^ (df = 1) = 20.8, *p* < 0.01). Sex was related to encountering olfactory cues (87.4 % of males vs. 64.3 % of females, *Χ*^2^ (df = 1) = 15.1, *p* < 0.001) as was employment (89.7 % of employed, 64.3 % of unemployed, and 78.2 % of students, *Χ*^2^ (df = 2) = 78, *p* < 0.05). Tobacco use status was also related to reporting olfactory cues, with all (100 %) the tobacco users having this experience compared to 69.1 % of the non-tobacco users (*Χ*^2^ (df = 1) = 26.9, *p* < 0.001).

From the MP data, slightly more than half of the sample (56.6 %, *n* = 116) reported that they had at least once been in the presence of pro-tobacco messaging. Of these 116 participants, 38 (32.8 %) reported such exposure one time and 78 (67.2 %) saw such messages two or more times. Seeing pro-tobacco messages most frequently happened in private settings (78.4 %, *n* = 91), less frequently outside or in other places (35.3 %, *n* = 41), and least frequently in a public setting (26.7 %, *n* = 31). Sex was related to encountering pro-tobacco messages (63.0 % of males vs. 44.3 % of females, *Χ*^2^ (df = 1) = 6.6, *p* < 0.05). Other demographics were not significant. Around three-quarters of the tobacco users (76.8 %, *n* = 53) compared to less than half of the non-tobacco users (46.3 %, *n* = 63) reported seeing such cues (*Χ*^2^ (df = 1) = 17.3, *p* < 0.001).

Most participants (76.6 %, *n* = 157) reported seeing anti-tobacco messages through at least one MP. Among those who saw these messages, around 21.0 % of them (*n* = 33) said they saw messages at just one MP, while 79.0 % (*n* = 124) of them had seen such messages two or more times. Anti-tobacco messages were encountered most frequently in private settings (82.2 %, *n* = 129), less frequently outside or in other places (47.8 %, *n* = 75), and least frequently in public settings (28.7 %, *n* = 45). No demographic variables were associated with seeing anti-tobacco messages. Tobacco use was related to seeing anti-smoking messages; interestingly, a higher percentage of tobacco users reported seeing anti-smoking messages compared to non-tobacco users (85.5 % vs. 72.1 %, *Χ*^2^ (df = 1) = 4.6, *p* < 0.05).

During the study period, most participants (88.3 %, *n* = 181) had been in a setting with known tobacco use restrictions, at least once when signaled with the MP. Among those who reported awareness of restrictions, only 17 (9.4 %) of them reported being in a place with tobacco restrictions once, and most of them (90.6 %, *n* = 164) reported awareness of restrictions more than once. Reporting restrictions of tobacco use occurred most frequently when one was in a private setting (85.1 %, *n* = 154), and less frequently in work/school setting (50.3 %, *n* = 91), outside or other places (44.8 %, *n* = 81), and public settings (40.3 %, *n* = 73). Neither demographic variables nor tobacco use status were associated with being in settings with restrictions. If we consider this subsample of those in areas with reported restrictions, 15.4 % (*n* = 28) were using tobacco themselves and 27.6 % (*n* = 50) could see another using tobacco at that moment.

### Results from End of Day (EOD)

Around a third of the participants (37.6 %, *n* = 77) reported tobacco use when they completed the EOD assessment, at least once during the 10-day study period. All participants who used tobacco reported smoking tobacco and about 41.6 % (*n* = 32) of them also reported using smokeless tobacco. Among the 77 people who used tobacco, slightly less than half (44.2 %, *n* = 34) only reported use at a single EOD, 14.3 % (*n* = 11) at two EODs, and 41.6 % (*n* = 32) at 3 or more EODs. Compared to the use of the overall tobacco, the use of smokeless tobacco was less frequent. Among 32 people who reported using smokeless tobacco from EOD assessment, most of them (75.8 %, *n* = 25) only reported use at a single EOD, two (6.3 %) people and five (15.6 %) people reported the use at two and three or more EODs, respectively. Through the EOD reports, only age was associated with self-reported use of tobacco; the average age for tobacco users was 26.3 (SD = 6.8) years while the age was 24.2 (SD = 5.6) years for non-tobacco users (*t* = -2.5, *p* < 0.05).

According to EOD assessments, most (76.1 %, *n* = 156) of the sample indicated that they had been in the presence of another tobacco user during the study period. Of these 156 participants, 51 (32.7 %) reported such exposure one time and 105 (67.3 %) reported two or more times. Notably, participants in Hyderabad (81.4 %, *n* = 96), had a higher percentage of reporting seeing presence of others using tobacco, compared to those from Kolkata (69.0 %, *n* = 60, *Χ*^2^ (df = 1) = 4.2, *p* < 0.05). A higher percentage of tobacco users were in the presence of other tobacco users compared to non-tobacco users (96.1 % vs. 64.1 %, *Χ*^2^ (df = 1) = 27.1, *p* < 0.01). About half (48.3 %, *n* = 99) of the participants reported being with other tobacco users in their immediate group, and more than half (57.1 %, *n* = 117) reported seeing other tobacco users in their view. Of these participants who reported other tobacco users in their immediate group, 34 (34.3 %) and 53 (45.2 %) participants reported the exposure once, respectively, and 65 (65.7 %) and 64 (54.7 %) two or more times, respectively. In the one analysis where females had higher exposure than males, more females (71.4 %, *n* = 50) reported in the EOD surveys that they had seen tobacco users in their view compared to males (49.6 %, *n* = 67, *Χ*^2^ (df = 1) = 8.9, *p* < 0.001). While no socio-economic differences were found for reporting presence of tobacco users in one’s immediate group, a higher percentage of tobacco users said there were other tobacco users in their immediate group compared to non-tobacco users (83.1 % vs. 27.3 %, *Χ*^2^ (df = 1) = 59.9, *p* < 0.001). Likewise, a higher percentage of tobacco users said that other tobacco users were in their view compared to non-tobacco users (71.3 % vs. 48.4 %, *Χ*^2^ (df = 1) = 10.4, *p* < 0.01).

Many of the participants (68.3 %, *n* = 140; 70.2 %, *n* = 144) reported exposure to visual and olfactory cues, respectively. Of those who were exposed to visual and olfactory cues, 42 (30.0 %) and 40 (27.8 %) reported the cues once, respectively, and 98 (70.0 %) and 104 (72.2 %) reported two or more times, respectively. No socio-economic characteristics were associated with visual or olfactory cues. A higher percentage of tobacco users were exposed to visual and olfactory cues compared to non-tobacco users, respectively (89.6 % vs. 55.5 %, *Χ*^2^ (df = 1) = 25.9, *p* < 0.001; 90.9 % vs. 57.8 %, *Χ*^2^ (df = 1) = 25.2, *p* < 0.001).

About one fourth (26.8 %, *n* = 55) of the participants saw pro-tobacco messages and slightly more than half (58.5 %, *n* = 120) saw anti-tobacco messages at least once during the 10-day study period. Of these participants who saw pro- and anti-tobacco messages, 26 (47.3 %) and 54 (45.0 %) reported seeing them one time and 29 (52.7 %) and 66 (55.0 %) saw reported seeing them two or more times. Males reported at a higher percentage seeing pro-tobacco messages compared to females (31.9 % vs.17.1 %, *Χ*^2^ (df = 1) = 5.1, *p* < 0.05). No socio-economic characteristics were found to be associated with seeing anti-tobacco messages. A higher percentage of tobacco users saw pro-tobacco messages compared to those who did not use tobacco (48.1 % vs. 14.1 %, *Χ*^2^ (df = 1) = 28.3, *p* < 0.01). Likewise, a higher percentage of tobacco users saw anti-smoking messages compared to non-tobacco users (80.5 % vs. 45.3 %, *Χ*^2^ (df = 1) = 24.6, *p* < 0.01).

### Sex versus tobacco use

Throughout the results, we often report that both sex and tobacco use were associated with exposure to social and environment cues. To determine which variable might be driving these findings, we separated the sample, and ran the analyses with both the MP and EOD data.

When we considered males and females separately, the significant differences between nontobacco and tobacco users remained. In contrast, when we divided the sample by tobacco use, we no longer observed significant differences in exposure to cues between males and females.

## Discussion

This research confirms the ubiquity of social and environmental cues about tobacco in India. Besides high rates of personal use of tobacco, the majority of study participants noted use by others, visual and olfactory cues, and messaging promoting tobacco use. Interestingly, there was also great awareness of anti-smoking messaging and restrictions regarding tobacco.

Considering demographics, sex was the only factor consistently associated with differential exposure. When we separated the analyses by sex we found the difference in cues was driven by the higher rates of personal tobacco use by males compared to females. Other socio-demographic factors, including age, education, and employment, were not related to social and environmental tobacco-related cues, suggesting that exposure reaches those of different demographic groups.

Reported tobacco use was related to most of the social and environment tobacco-related cues, assessed through both the MP and EOD assessments in this study. Tobacco-related exposures were more often reported by tobacco users compared to non-tobacco users, suggesting potential influences on tobacco use. The only exception was restrictions to tobacco use. We found restrictions of tobacco use, noted by 88 % of the participants, were not related to tobacco use status, signifying that although restrictions of tobacco use were very prevalent in these two Indian cities, they were equally observed by both tobacco users and non-tobacco users. Compliance with restrictions is of concern, as this data shows that even in the presence of perceived rules, personal and others use of tobacco was occurring.

Private places (i.e., homes and cars) and outside were the most prevalent settings for participants to use tobacco or to be exposed to tobacco-related cues and messages. Interestingly, private places, instead of public settings and school and workplaces, were also the most common setting for participants to report restrictions of tobacco use. This may reflect self-imposed policies, such as banning tobacco use in personal homes and cars. Conversely, public settings (e.g., bars, restaurants, shopping malls, and places of worship, etc.) and schools and workplaces were usually less common settings for participants to use tobacco or report exposures. Tobacco use was substantially more likely to happen when participants were with friends and coworkers, compared to when alone or with families. Consistent with studies conducted in the U.S. and other developed countries, this research found that exposure to others using tobacco was common and related to personal tobacco use [13, 14]. This phenomenon of “social smoking” [13, 14, 37] appears as a risk factor for tobacco use in India. This study also shows that it is not just cigarette smoking, as participants were aware of others using smokeless tobacco. This may be particularly important to explore with future efforts to curb smokeless tobacco use in India [38].

This study was the first EMA study in a LMIC to detail exposure to social and environmental exposures. Using the innovative approach of EMA, this study supports the hypothesis that reported tobacco use is associated with social and environmental cues and messages including the presence of other individuals using tobacco, the individuals’ public settings, and the visual and olfactory cues of tobacco, etc. The study has several key strengths. First, EMA methods and new technology were used to collect data in real time, thus limiting systematic recall bias. The study also collected short-term recall data to compare against the momentary data. Both approaches resulted in similar association for tobacco use and exposures to cues, and we feel that the EOD approach may serve as a proxy and limit research burden associated with MP [39]. Additionally, this is the first research combining the use of smokeless tobacco into an EMA studying the antecedents of tobacco use. Previous studies have only focused on “smoking” as the behavioral outcome. Our findings, however, resemble those from a recent study [2]. In this work that collected self-report retrospective data on tobacco use throughout the country, the prevalence rate for tobacco use was 34.6 %. Around a fifth (20.6 %) consumed only smokeless tobacco, 8.7 % only smoked only, and 5.3 % used both.

Like studies conducted in other settings, our results show that environmental cues were frequent and significantly related to tobacco use [9, 17]. To our knowledge, this was the first study using an EMA to assess the relationship of the exposure to tobacco-related messages with real-time tobacco use. Previous EMA research has found that seeing tobacco was associated with intentions to use tobacco and future risks of using tobacco products, [19, 31, 40] instead of actual tobacco use behavior. Retrospective research has demonstrated strong relationships between media/message exposure promotion tobacco with initiation and use; [41, 42] however, all these studies may be subject to recall and self-report bias.

Our study had several limitations. First, the study sample does not represent the overall population in India due to our non-random sampling method and focus on the cities of Hyderabad and Kolkata. To explore potential differences, we purposely recruited tobacco users as well as non-users. Also, participants were from urban settings in India, thus limiting the generalization to rural areas where tobacco use patterns and predictors may differ [2]. The fact that the average age of our sample was 25.0 (SD = 6.2) also limited the study generalization to a comparatively young population in India. Additionally, since owning an Android phone was one of the eligibility criteria, our sample might be skewed towards higher socio-economic group and comprised a proportion of population of mobile owners in India. However, this bias is less of a concern due to the following facts. India has an estimated 117 million smartphone users [43] and, as of September 2014, over 930 million mobile phones, reflecting ownership by over 77 % of the country’s population [44]. It is also worth noting that inexpensive “bootleg” versions of mobile phones and Android phones were widespread in our sample and study sites. Thus, this limitation may be relatively minor.

This study validity depended profoundly on participant compliance, while our MP compliance was low (.46). The study results would be largely biased if the participants who were more compliant actually were exposed to social and environmental exposures differently than those who were less compliant. However we did not find this to be true since only employment is the only predictor of the compliance rates [35] and the employment status did not actually predict the social and environmental exposures; and none of our examined exposures were significantly related to the MP or EOD compliance rates. Regardless, future studies should improve EMA study compliance to achieve more satisfactory internal validity. We also acknowledged that the percentage of participants who reported social and environmental exposures measured by EOD assessment was generally higher than that of MP assessment, indicating the importance of a future exploration on the differences of using these two methods to capture tobacco-related cues. This discrepancy can be attributed to the fact that participants failed to complete a MP survey while distracted by some social and environmental cues (e.g., chatting with smoking friends in a shopping mall), but still captured these daily cues in the EOD survey. This assumption needs to be further validated by future studies that explore EMA compliance by examining the specific moments that lead to non-compliances. This study was also limited in its reliance on self-report data [45]. Particularly, self-reported data are subject to individuals’ momentary mood and can be adversely influenced by deception or self-deception [24]. Other objective measurements of tobacco use and cues might lead to different conclusions. Nevertheless, EMA records of cigarette consumption has proven in other settings to correlate well with the biomedical marker carbon monoxide (CO) [25]. More studies are require to validate the use EMA for testing real-time smokeless tobacco use, as we believe this is the first to explore this behavior.

## Conclusions

Future cessation interventions and advocacy efforts must address the pervasive nature of tobacco in India. This study found that participants frequently encountered others who were using tobacco as well as visual and olfactory cues. As these might be strong risk factors for personal tobacco use, there should be attempts to lessen public use. Better enforcement of restrictions and bans on tobacco advertising and promotion are needed, as this work shows that Indians are seeing such tobacco related cues.

This work provides great support for employing real-time and individualized ecological momentary approaches [46] in natural environments and interventions. By using the predictive algorithms programmed in the EMI system, there can be on the spot psychological and behavioral guidance to lessen the impact of social and environmental cues for tobacco use. If individuals receive timely and contextually relevant interventions to respond and cope with encountered cues, they might be better able to reduce or quit tobacco use. Applications that help individuals quit smoking have been implemented in some western countries such as New Zealand and in the United States [47, 48] with highly successful outcomes; they have not yet, to our knowledge, been employed in LMICs. Our study shows that implementing research studies in India using EMA methods are important and feasible; we are hopeful that similar studies can be successfully implemented in India or other similar LMICs to curb or eliminate tobacco use.

## Abbreviations

EMA: ecological momentary assessment
EOD: end of day
LMIC: lower- or middle-income country
MP: momentary prompt
